# Six Hours after Infection, the Metabolic Changes Induced by WSSV Neutralize the Host’s Oxidative Stress Defenses

**DOI:** 10.1038/srep27732

**Published:** 2016-06-09

**Authors:** I-Tung Chen, Der-Yen Lee, Yun-Tzu Huang, Guang-Hsiung Kou, Han-Ching Wang, Geen-Dong Chang, Chu-Fang Lo

**Affiliations:** 1 Institute of Bioinformatics and Biosignal Transduction, College of Bioscience and Biotechnology, National Cheng Kung University, Tainan 701, Taiwan; 2 Department of Life Science, National Taiwan University, Taipei 106, Taiwan; 3 Technology Commons, National Taiwan University, Taipei 106, Taiwan; 4 Department of Applied Chemistry, National Chiayi University, Chiayi 600, Taiwan; 5 Institute of Biotechnology, College of Bioscience and Biotechnology, National Cheng Kung University, Tainan 701, Taiwan; 6 Graduate Institute of Biochemical Sciences, National Taiwan University, Taipei 106, Taiwan; 7 Center for Systems Biology, National Taiwan University, No. 1, Section 4, Roosevelt Road, Taipei 106, Taiwan

## Abstract

Levels of intracellular ROS (reactive oxygen species) were significantly increased in hemocytes collected from WSSV-infected shrimp within the first 30–120 min after infection. Measurement of the NADPH/NADP^+^ and GSH/GSSG ratios revealed that after a significant imbalance toward the oxidized forms at 2 hpi, redox equilibrium was subsequently restored. Meanwhile, high levels of lactic acid production, elevated NADH/NAD^+^ ratios, and metabolic changes in the glycolysis pathway show that the Warburg effect was triggered by the virus. The timing of these changes suggests that WSSV uses this metabolic shift into aerobic glycolysis to counteract the high levels of ROS produced in response to viral infection. We further show that if the Warburg effect is inhibited by chemical inhibition of the PI3K-Akt-mTOR signaling pathway, or if the pentose phosphate pathway is chemically inhibited, then in both cases, the production of intracellular ROS is sustained. We conclude that WSSV uses the PI3K-Akt-mTOR-regulated Warburg effect to restore host redox balance and to counter the ROS produced by the host in response to WSSV infection. We also found that pyruvate kinase activity was inhibited by WSSV. This inhibition is likely to increase the availability of the raw materials essential for WSSV gene expression and replication.

Reactive oxygen species (ROS) are highly reactive, unstable chemical molecules that, in addition to their other various roles in cellular biological functions, are also an important part of the host’s immune defenses against an invading pathogen. An over-accumulation of ROS or a deficiency of antioxidants leads to an imbalance in the redox state and causes oxidative stress in the cell. However, although an excess of oxidizing species destroys a cell’s macromolecules, the oxidative stress that is induced in response to virus infection benefits the host by limiting virus replication^1^,^2^. For example, in a previous study, we found that the DNA binding activity of the important WSSV transcriptional regulator IE1 was inhibited under oxidative conditions^3^.

The production of ROS is thus an important cellular response that provides an infected shrimp with immediate protection. Curiously, however, although large quantities of oxidants are produced by shrimp hemocytes after challenge with *Vibrio spp.*^4^,^5^, when shrimp are infected by white spot syndrome virus (WSSV), ROS are only detected at the very late stages of infection (24 hours post injection), while at the viral replication stage (~12 hpi) the ROS levels are actually lower in the WSSV infected shrimp than in the controls^3^,^6^,^7^,^8^. This suggests either that for some reason the host is not producing ROS in response to WSSV infection or else that WSSV is deploying effective countermeasures against the host’s ROS defenses in order to achieve replication. In fact, there is already evidence that WSSV is counteracting the cell’s ROS defenses. For instance, WSSV appears to restore the cell to a reduced state by boosting the antioxidant enzyme activity of superoxide dismutase (SOD) and glutathione peroxidase (GPx)^7^.

There is also evidence that at the viral replication stage, WSSV induces metabolic changes that are characteristic of the Warburg effect^6^. The Warburg effect describes the transitory changes in the metabolism that occur in cancer cells, proliferating cells, and certain virus-infected cells^9^,^10^,^11^,^12^,^13^. The metabolic changes in these cells indicate that some glucose is diverted into lactic acid production instead of ultimately being catabolized by the tricarboxylic acid (TCA) cycle. This re-routing is a defining characteristic of the Warburg effect. It occurs in the presence of oxygen and is known as aerobic glycolysis. This altered metabolism enables the cell to meet the increased demand for energy and for the building blocks needed for macromolecule biosynthesis. It also allows the cell to maintain its redox state by supplying sufficient quantities of reduced nicotinamide adenine dinucleotide phosphate (NADPH), which is a major cellular reducing agent that is mostly produced by the pentose phosphate pathway (PPP)^14^,^15^,^16^. Su *et al*.^17^ further demonstrated that since WSSV gene expression, genome replication and several characteristics of the Warburg effect, including the accumulation of lactic acid, were all decreased by chemical inhibition of the PI3K-Akt-mTOR pathway, then the WSSV-induced Warburg effect must be controlled by this pathway.

In the present study, we investigate both the timing and the mechanisms by which WSSV alters its host’s metabolism to restore host redox balance and to counter the immediate increase in ROS production that occurs after WSSV infection. In order to build a more complete model, the time-series data that we present here includes early time point (0.5, 1, 2 and 6 hpi) that have not previously been extensively analyzed. We used a confocal spectral microscope and flow cytometry analysis to intensively measure the ROS levels in shrimp during the first WSSV replication cycle (ie. during the first 24 h of infection). The redox state in WSSV-infected shrimp stomach cells was also determined by using LC-ESI-MS analysis to monitor the ratio of reduced nicotinamide adenine dinucleotide phosphate to nicotinamide adenine dinucleotide phosphate (NADPH/NADP^+^) and reduced glutathione to oxidized glutathione (GSH/GSSG). We also used an *in vivo* shrimp metabolomics platform to monitor the characteristic changes induced by the Warburg effect in shrimp after WSSV infection. These changes include the accumulation of lactic acid and the elevated glucose metabolism activity that occurs during the virus genome replication cycle. Changes in the activity of five key enzymes that are involved in the Warburg effect and the TCA cycle were also monitored. Lastly, *in vivo* chemical inhibition of the PI3K-Akt-mTOR pathway and the pentose phosphate pathway was used to further elucidate how the WSSV-induced Warburg effect acts to neutralize the oxidative stress caused by the increase in ROS production.

## Results

### WSSV induces high levels of ROS production in shrimp hemocytes at the initial stage of infection (0.5~2 hpi)

**Figure 1:**
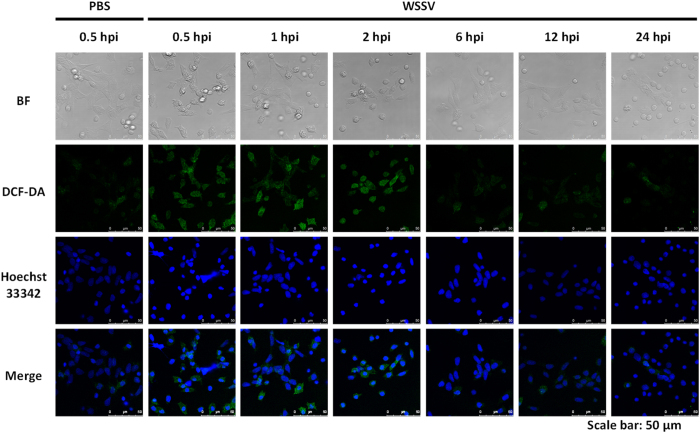
ROS accumulated in shrimp hemocytes in the first 2 h of WSSV infection. The hemocytes from WSSV-infected and control (PBS injected) shrimp were stained with dichlorodihydrofluorescein diacetate (DCF-DA) to reveal the intracellular levels of ROS. Hoechst 33342 was used to counterstain the nucleus.

In this study, we used the fluorescent indicator 2′,7′-dichlorofluorescein (DCF) to monitor the levels of intracellular ROS in hemocytes collected from WSSV-infected and control shrimp. The results showed that WSSV infection transiently induced high levels of ROS production at the initial stage of infection from 0.5 hpi to 2 hpi (Fig. 1). Subsequently, after 6 hpi, the DCF fluorescence signal was reduced to the baseline level, indicating a return to a lower oxidative state in the cells as the viral infection progressed (Fig. 1).

**Figure 2:**
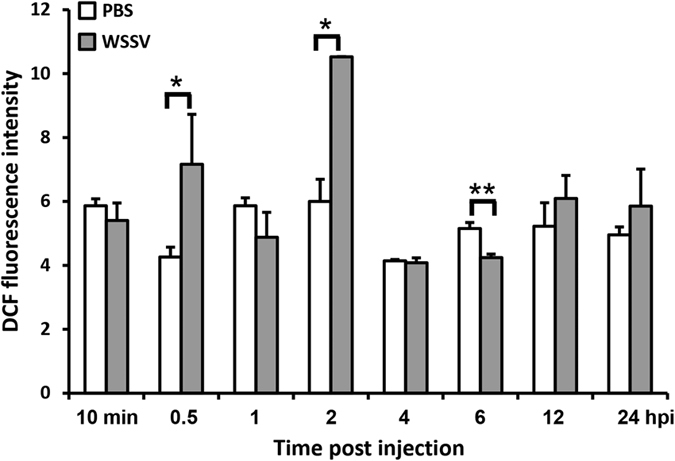
The intracellular ROS levels in the shrimp hemocytes after DCF staining were quantified by flow cytometry scanning. Each bar indicates the mean ± SD of DCF fluorescence intensity in 30,000 hemocytes collected from WSSV- or PBS-injected shrimp. Single (P < 0.05) and double (P < 0.005) asterisks indicate a statistically significant difference between the experimental and control groups.

Quantification of the fluorescent DCF intensities by flow cytometry revealed a similar pattern (Fig. 2). The additional time points at 10 min and 4 hpi in Fig. 2 suggest that extra ROS production only occurs during the 0.5~2 hpi time period. The return to control levels at 1 hpi may reflect actual changes in production of ROS in the infected cell because while the DCF signal in Fig. 1 monitors the accumulated level of ROS, the flow cytometry used in Fig. 2 provides a better reflection of the ongoing dynamic regulation of the infected cells’ redox balance Fig. 2 therefore implies that changes in the state of the cells might in fact lead to a temporary pause in ROS production at around 1 hpi.

### After the initial 2 h period of oxidative stress, higher levels of NADPH and GSH restore the cellular redox balance

To monitor the intracellular redox status in shrimp from the early stage to the late stage of WSSV infection, we used an LC-ESI-MS system to measure the ratio of the reducing agent NADPH to its respective oxidizing agent, NADP^+^. Since NADPH is a low abundance metabolite, for this experiment we used a different tissue (stomach) and a better extraction method to obtain a cleaner signal; for details, please see materials and methods. We also used LC-ESI-MS to measure the ratio of the reduced form of glutathione (GSH) to the oxidized form (GSSG). The total amount of glutathione (GSH + 2 x GSSG) was also recorded.

**Figure 3:**
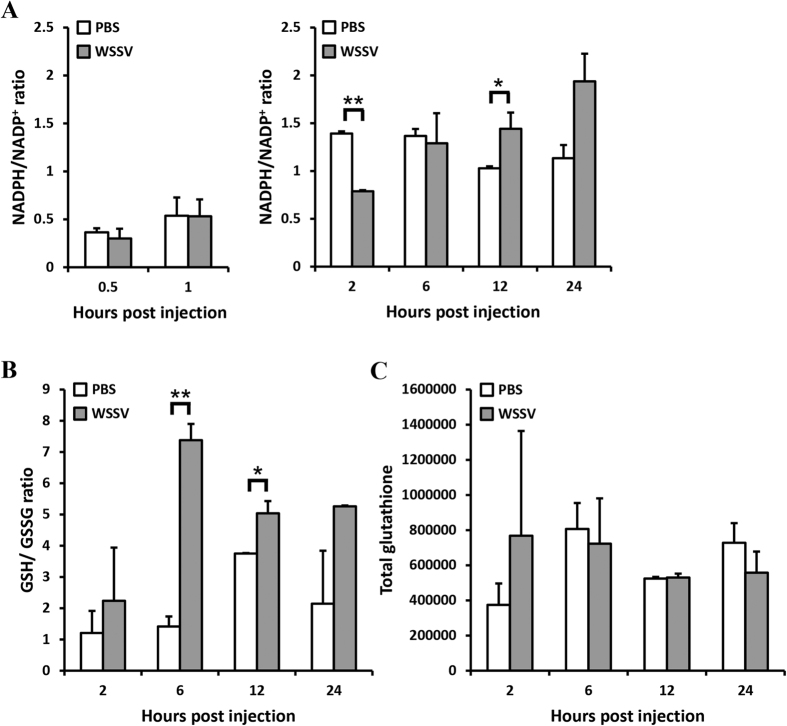
Loss of redox balance in the stomachs of WSSV-infected shrimps. Each bar indicates the ratio ± SD of (**A**) NADPH/NADP^+^ and (**B**) GSH/GSSG and (**C**) total glutathione (i.e. GSH + 2x GSSG) in five sets of pooled samples of stomachs collected from PBS- or WSSV-injected shrimp at the indicated time points. The data in (**A**) is show in two separate groups because it was obtained from two separates independent runs of the same experiment. Single (P < 0.05) and double (P < 0.005) asterisks indicate a statistically significant difference between the experimental and control groups.

A significantly lower ratio of NADPH/NADP^+^ was observed at 2 hpi (Fig. 3A) indicating that there was oxidative stress in shrimp stomachs at the initial stages of WSSV infection. This is consistent with the results shown in Figs 1 and 2.

Subsequently, we observed an increase in both ratios starting from 6~12 hpi (Fig. 3A,B). High levels of NADPH would drive a downstream increase in GSH^18^, and an increase in both of these reducing forces would act to neutralize the ROS in the WSSV-infected cells. We therefore conclude that these elevated levels of NADPH and GSH are the reason for our previous observation that ROS concentrations return to normal after 2 h.

### Additional evidence that the Warburg effect had been triggered in WSSV-infected shrimp: production of lactic acid and an increase in the NADH/NAD^+^ ratio

**Figure 4:**
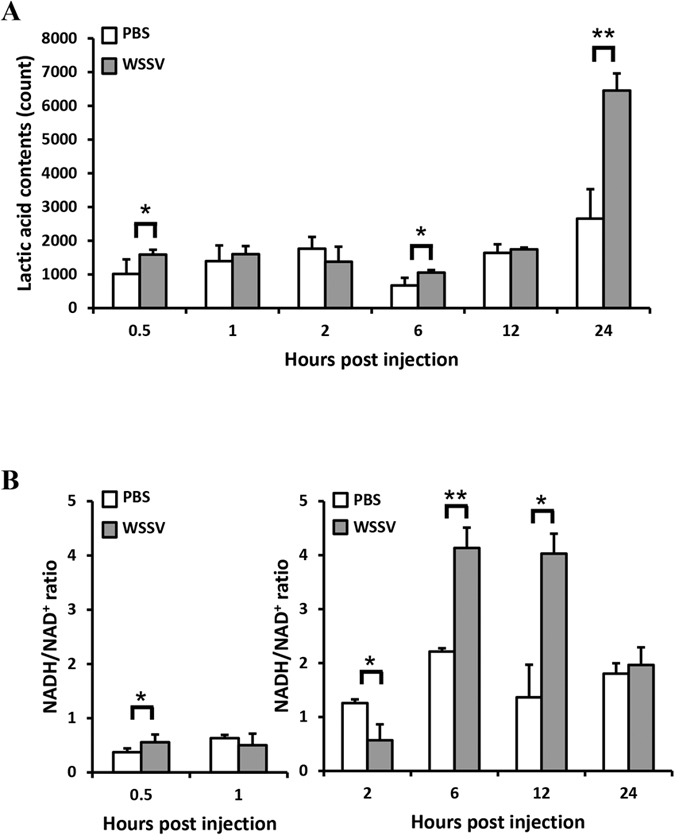
Lactic acid accumulation and changes in the NADH/NAD^+^ ratio in WSSV-infected shrimp. (**A**) Lactic acid content in shrimp plasma after challenge with WSSV. (**B**) The NADH/NAD^+^ ratio in pooled samples of shrimp stomachs after WSSV challenge. Each bar indicates the mean ± SD for five sets each of the WSSV-challenged shrimps and the PBS-injected controls at the indicated time points. The data in (**B**) is show in two separate groups because it was obtained from two separates independent runs of the same experiment. Single (P < 0.05) and double (P < 0.005) asterisks indicate a statistically significant difference between the experimental and control groups.

In cancer cells, the Warburg effect detoxifies ROS and maintains the redox balance by rerouting the glucose metabolism through the PPP to generate NADPH^14^,^19^. We have already shown that NADPH levels are increased (Fig. 3A), and next we looked for more evidence that the Warburg effect had been triggered. Specifically, we looked for two other changes that the Warburg effect would be expected to produce. Increased accumulation of lactic acid is one of these important indicators, and, as expected, we found that the lactic acid levels in the plasma of WSSV-infected shrimp were significantly higher than the levels in control shrimp at 6 and 24 hpi (Fig. 4A). Unexpectedly, however, we found that lactic acid levels were also significantly higher at 0.5 h after infection, i.e. at a time that was previously thought to be too early for the Warburg effect to have been induced. The second indicator is the ratio of reduced nicotinamide adenine dinucleotide (NADH) to nicotinamide adenine dinucleotide (NAD^+^)^20^. These results were more in line with our expectations: for consistency, in this experiment we again used stomach cells, and found that the NADH/NAD^+^ ratio was also significantly elevated at 6 and 12 hpi (Fig. 4B). Taken together these data are consistent with our hypothesis that WSSV induces the Warburg effect in WSSV-infected cells.

Since the Warburg effect alters the cell’s metabolism, we next used an *in vivo* shrimp metabolomics platform (please see Supplementary data) to further investigate changes in the cell’s metabolomic activity during the course of WSSV infection. Some of these results are presented as supplementary data while the changes in the glycolytic pathway are presented below.

### More evidence that the Warburg effect had been triggered: elevated glycolysis activity in WSSV-infected cells

**Figure 5:**
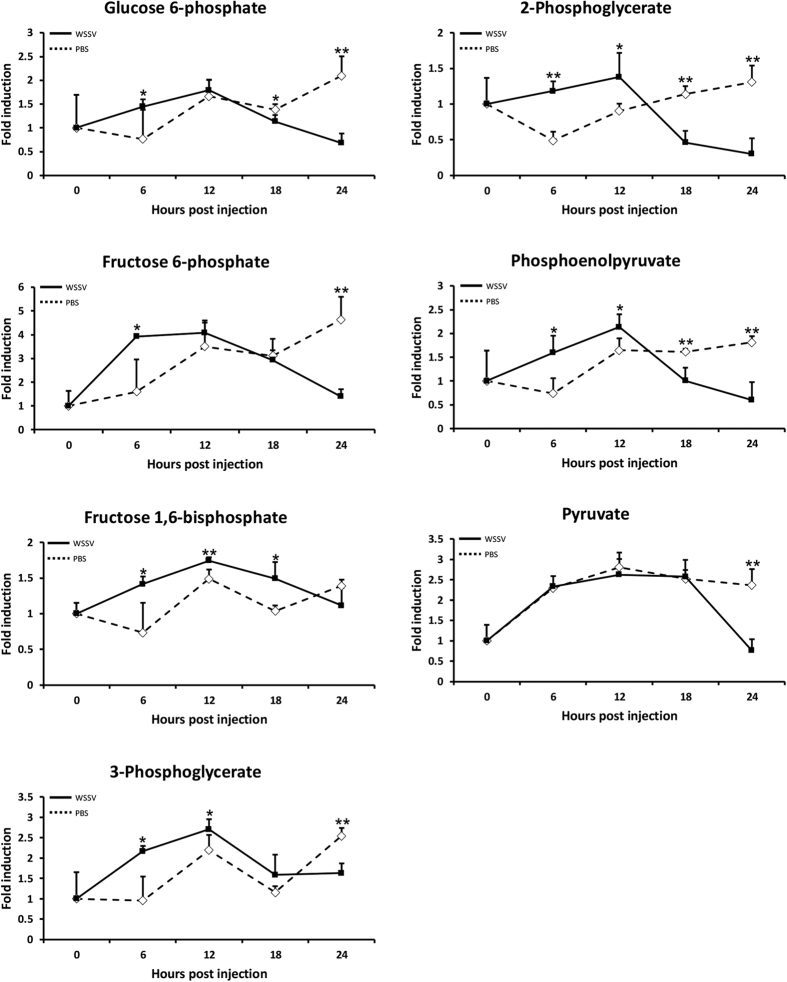
Measurement of the levels of seven glycolytic metabolites in the hemocytes of WSSV-infected shrimp. An LC-ESI-MS metabolomics platform was used to monitor the levels of several glycolytic intermediates during WSSV infection. Each value represents the mean level (n = 5) of the indicated glycolytic metabolite relative to its level at 0 hpi. Single (P < 0.05) and double (P < 0.005) asterisks indicate a statistically significant difference between the experimental and control groups.

Changes in the glycolytic pathway metabolites in WSSV-infected and control (PBS) shrimp hemocytes are shown in Fig. 5. (Although only hemocyte results are presented here, stomach cells and hemocytes are both targeted by WSSV, and the WSSV-induced metabolomic changes in both of these cell types are very similar [compare Fig. 5 and Sup Fig. 3]). After WSSV infection, there was a noticeable increase in the levels of five of the measured glycolytic intermediates – fructose 1,6-bisphosphate (FBP), 3-phosphoglycerate (3-PG), 2-phosphoglycerate (2-PG) and phosphoenolpyruvate (PEP) – especially at 6~12 hpi. These increases in glycolytic activity are consistent with the metabolic changes that the Warburg effect is expected to produce. Interestingly however, although PEP levels were elevated, the level of pyruvate, which is the end product of glycolysis, was no higher than in the corresponding controls (Fig. 5). The metabolism from PEP to pyruvate is one of the critical regulatory points for glycolysis, and this reaction is catalyzed by pyruvate kinase. In the next section of this study, we therefore monitored the activity of this enzyme as well as four other key regulating enzymes.

### Changes in the activity of enzymes related to the Warburg effect after WSSV infection

**Figure 6:**
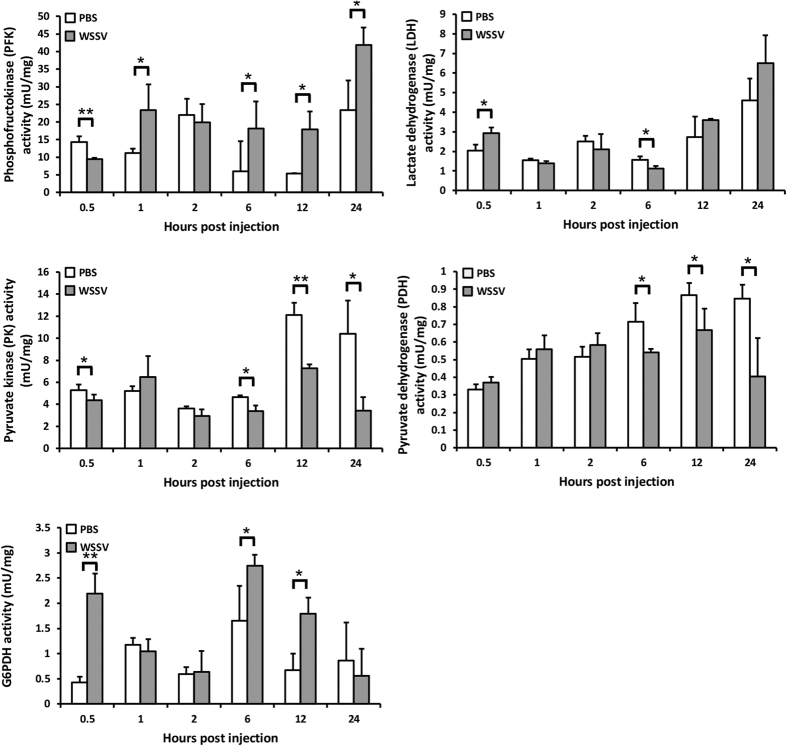
Measurement of activity levels of enzymes related to the Warburg effect in WSSV-infected shrimp hemocytes. Each bar indicates the mean ± SD activity of the indicated enzymes in five replicated sets of shrimp hemocytes collected from PBS- or WSSV-injected group at the indicated times. Single (P < 0.05) and double (P < 0.005) asterisks indicate a statistically significant difference between the experimental and control groups.

Enzyme activity changes were monitored in five key regulatory enzymes that are involved in the Warburg effect and the TCA cycle: phosphofructokinase (PFK), pyruvate kinase (PK), Glucose-6-phosphate dehydrogenase (G6PDH), lactate dehydrogenase (LDH) and pyruvate dehydrogenase (PDH). As Fig. 6 shows, except at 2 hpi, the enzyme activity of PFK was significantly increased in the hemocytes of WSSV-infected shrimp from 1 to 24 hpi. While this is consistent with our previous observations of elevated aerobic glycolysis during virus replication, these results also suggest that the Warburg effect might in fact to be induced much earlier than 12 hpi as our previous model suggested. Similarly, we also found that the enzyme activity of pyruvate kinase was significantly reduced in the hemocytes of WSSV-infected shrimp from 6 to 24 hpi. Since the final step of glycolysis is the irreversible conversion of PEP to pyruvate in a reaction which also produces a single molecule of ATP (adenosine triphosphate), the reduced activity of this enzyme explains why PEP and the other upstream metabolites in Fig. 5 were accumulated while the level of pyruvate remained low. The G6PDH results, which show significantly increased enzyme activity at 0.5, 6 and 12 hpi, and also broadly consistent with the idea that the Warburg effect might be induced relatively soon after infection. Meanwhile, the enzyme activity of pyruvate dehydrogenase was continuously inhibited in the hemocytes of WSSV-infected shrimp from 6 to 24 hpi. Taken together, these results suggest that the start time for the Warburg effect might be around 6 hpi after all. From this time onward, the low activity of PDH implies that the conversion of pyruvate to acetyl-CoA was limited, which would mean that glucose is diverted into lactic acid production instead of being used in the TCA cycle.

Lastly, the relative stability of the LDH data suggests that the increased levels of lactic acid (Fid 4A) must be due to increased metabolic throughput rather than any sustained increase in LDH activity.

### The PI3K-Akt-mTOR-regulated Warburg effect limits ROS production in shrimp

**Figure 7:**
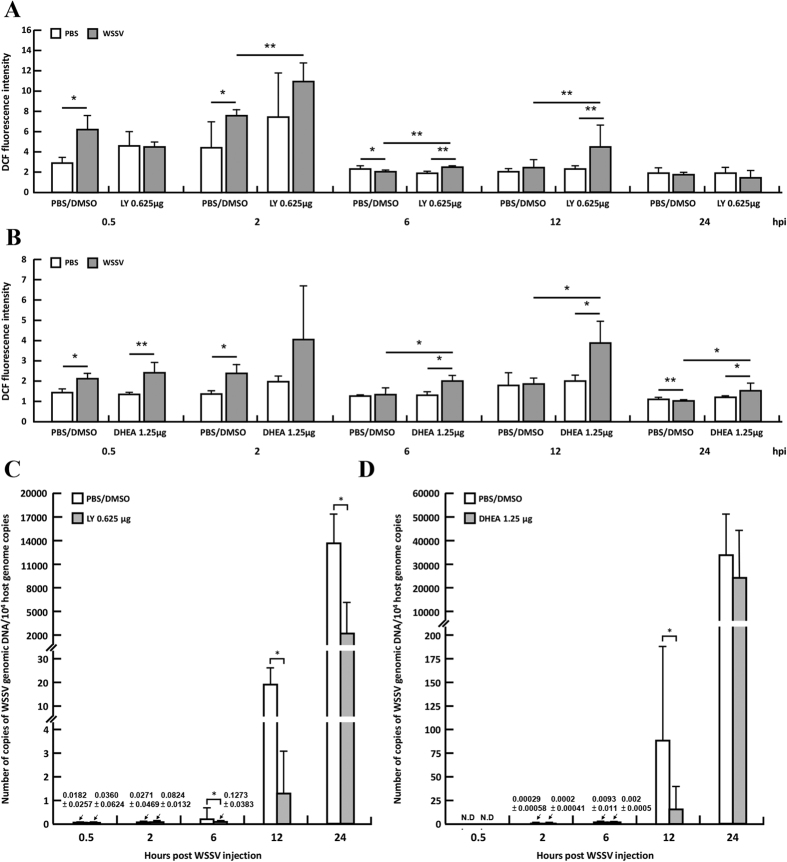
Changes in intracellular ROS levels and WSSV genome copy number after chemical inhibition of the PI3K-Akt-mTOR pathway with LY294002 and inhibition of G6PDH activity with DHEA. Hemocytes were collected from shrimp that had been pretreated with (**A**,**C**) LY294002/DMSO or PBS/DMSO (control) or (**B,D**) DHEA/DMSO or PBS/DMSO (control), and then injected with WSSV or PBS. (**A,B**) The ROS levels in shrimp hemocytes were quantified by flow cytometry scanning. Each bar indicates the mean ± SD for the WSSV-challenged shrimps and the PBS-injected controls at the indicated time points. (**C,D**) Each bar indicates the mean ± SD of the number of copies of WSSV genomic DNA in the WSSV-injected shrimp pretreated with (**C**) LY294002 or (**D**) DHEA. Single (P < 0.05) and double (P < 0.005) asterisks indicate a statistically significant difference between the experimental and control groups.

In this experiment, ROS production was monitored in shrimp that had been pre-treated with a chemical inhibitor of the PI3K-Akt-mTOR signaling pathway, LY294002, and then challenged with WSSV. In shrimp that had been pretreated with PBS/DMSO vehicle only, WSSV infection initially led to ROS levels that were significantly higher than in the uninfected shrimp. These elevated ROS levels were only seen from 0.5 hpi to 2 hpi; thereafter, from 6~24 hpi, ROS levels in the infected shrimp were either the same or lower than in the unchallenged controls (Fig. 7A). By contrast, after pretreatment with the chemical inhibitor LY294002, from 2~12 hpi, ROS levels in the WSSV-infected shrimp continued to be significantly higher than in the WSSV group that had been pretreated with PBS/DMSO vehicle only. We also found that WSSV genome replication was suppressed after the PI3K-Akt-mTOR pathway was inhibited by LY294002 (Fig. 7C). Since chemical inhibition of the PI3K-Akt-mTOR signaling pathway resulted in sustained production of intracellular ROS, and since inhibition of this pathway is also known to suppress the WSSV-induced Warburg effect^17^, we therefore conclude that the PI3K-Akt-mTOR-regulated Warburg effect acts to limit ROS production in WSSV-infected shrimp.

### DHEA inhibition of G6PDH activity leads to elevated levels of ROS in WSSV-infected shrimp

The increased enzyme activity of G6PDH from 6~12 hpi after WSSV infection (Fig. 6) would lead to the production of NADPH, which in turn would neutralize the ROS produced by the shrimp. Here, we used DHEA to specifically inhibit G6PDH activity and then monitored changes in ROS levels and virus replication. In shrimp that had been pretreated with DHEA, WSSV infection led to significantly higher ROS levels than in PBS controls at 0.5 hpi and from 6–24 hpi (Fig. 7B). The data further shows that from 6–24 hpi, ROS levels were significantly higher in DHEA-inhibited WSSV-infected shrimp than in vehicle-only WSSV-infected shrimp. However, we also found that virus genome replication in the DHEA-pretreated shrimp was significantly lower only at 12 hpi, and not at 24 hpi. Interestingly, this result is consistent with the activity data shown in Fig. 6, which similarly suggests that G6PDH activity may no longer be important for virus replication at 24 hpi. This idea is further supported by the relatively low levels of accumulated ROS in all of the shrimp at 24 hpi (DCF fluorescence intensity ranges from 1~1.5; Fig. 7B), which suggests that it is no longer necessary for the virus to maintain redox balance at this time.

## Discussion

Our results here show that within 30 min~2 h after WSSV injection, ROS levels were significantly increased in shrimp hemocytes (Figs 1 and 2). We interpret this to mean that WSSV induced transient ROS production in the initial stage of infection, and that this represents an immediate immune response from the shrimp. This is the first time that shrimp have been shown to increase their ROS levels in response to WSSV. Subsequently, from ~4 hpi to the end of the experiments at 24 hpi, ROS levels returned to normal, which is consistent with previous observations of shrimp ROS production after WSSV infection^6^. Very few other viruses appear to be capable of neutralizing their host cell’s ROS defenses; one rare example is the human cytomegalovirus (HCMV)^21^.

In general, both ROS neutralization and the intracellular reduction-oxidation (redox) balance are chiefly mediated by the reducing agent NADPH. NADPH plays an important role in regenerating the cellular antioxidant capacity by providing a reducing force for glutathione reductase (GR), thioredoxin reductase (TrxR) and catalase (CAT), which are the three critical enzymes in the ROS detoxification system^18^. Measurement of the ratios of NADPH/NADP^+^ and GSH/GSSG in shrimp after WSSV infection revealed a significant imbalance toward NADP^+^ at 2 hpi (Fig. 3A), ie at the same time as the elevated ROS levels (Figs 1 and 2). Subsequently the ratio of reductants to oxidants return to normal, and at 6 hpi and 12 hpi, the ratios were even higher than in the control shrimp (Fig. 3A,B). These data show that there is a strong correlation between the redox state and ROS production, and further suggest that WSSV has developed a powerful and early-acting mechanism to combat the cell’s ROS defenses.

NADPH is produced in higher quantities when glucose metabolism is diverted through the pentose phosphate pathway (PPP)^22^. This re-routing of glucose metabolism is one of the first changes that occur in response to high levels of ROS. The transition from normal glucose metabolism to aerobic glycolysis is known as the Warburg effect. Previously, we found that WSSV induced Warburg-like changes in the metabolic pathway at the genome replication stage of infection (~12 hpi)^6^. These changes imply that the WSSV-infected cell would not only consume large quantities of nutrients and maintain high levels of anabolism, but also increase lactic acid production. Here, as in our previous study, we found lactic acid production was increased in shrimp as early as ~6 h after WSSV infection (Fig. 4A), and further, that NADH was also increased at 6~12 hpi (Fig. 4B). These changes suggest that the Warburg effect is driving the high levels of NADH production in the cell, and that WSSV is inducing aerobic glycolysis during replication. This benefits the virus because although aerobic glycolysis is a less efficient way of generating energy, it produces ATP at a higher rate^16^,^23^. At the same time, the increased lactic acid production indicates that glycolytic intermediates are rerouted into other pathways so as to help maintain adequate supplies of the macromolecules needed for biosynthesis. Thus, for example, re-routing into the PPP supports nucleic acid biosynthesis.

Pyruvate is the end product of glycolysis, and its conversion to lactic acid by lactate dehydrogenase (LDH) also causes one molecule of NADH to be converted to NAD^+^. Normally, the accumulation of metabolic intermediates causes glycolysis to shut down, but high levels of NAD^+^ override this feedback inhibition mechanism, and force the glucose metabolism to continue. This forcing also causes NAD^+^ to be converted back to NADH. Lactic acid production therefore drives the increase in aerobic glycolysis as well as driving the increased production of NADH^12^. As noted above, the ratio of NADH/NAD^+^ in WSSV infected shrimp was significantly elevated at 6 and 12 hpi (Fig. 4B) and this runs in parallel to the increase in lactic acid production (Fig. 4A). Similar observations have been reported in cancer cells^24^.

Our metabolomics analysis further confirmed that glycolysis activity in shrimp was up-regulated starting at ~6 hpi after WSSV infection (Fig. 5). Production of most of the glycolytic intermediates was increased, with the exception of pyruvate. Meanwhile, the enzyme activity of phosphofructokinase (PFK), which phosphorylates fructose 6-phosphate in glycolysis, was significantly increased after WSSV infection from 1 hpi (Fig. 6). Pyruvate is converted from PEP by the glycolytic enzyme pyruvate kinase (PK), and after WSSV infection, we found that levels of PEP were increased at the same time as the sustained decrease in pyruvate (Fig. 5). This suggests that PK enzyme activity is affected by the virus, and Fig. 6 confirms that the PK enzyme activity was significantly inhibited from 6 hours post WSSV infection.

Unfortunately, our knowledge of shrimp PK is still limited, and there is no obvious correspondence between the three shrimp PK isoforms and the four PK isoforms found in mammalian cells (PKL, PKR, PKM1, and PKM2)^25^. In mammal, cancer cells express the M2 isoform of PK^26^, and although PKM2 is less efficient at catabolizing ATP and PEP to pyruvate, its activity is more easily regulated by the energy molecules, upstream glycolytic intermediates, and growth factors. Inhibition of PKM2 causes the upstream metabolites to accumulate and then become re-routed into subsidiary pathways such as the PPP to produce the building blocks necessary for macromolecule biosynthesis and cancer cell proliferation^19^. In this way, cancer cells are able to regulate glycolysis in an efficient manner to maintain a balance between the production of energy (oxidative phosphorylation) and biomass production. In shrimp, however, there is no PK isoform that is equivalent to PKM2, and there is not yet any evidence to suggest that shrimp PK is being regulated by the same mechanisms. More work will therefore be needed to discover how the virus is able to regulate the activity of the shrimp PKs.

Inhibition of PK activity increases the concentration of upstream glycolytic intermediates, and as noted above, these intermediates are redirected into alternative glycolytic pathways, such as the PPP. We have shown previously that activity of the key enzyme of the PPP, glucose 6-phosphate dehydrogenase (G6PDH), was increased at the viral genome replication stage^6^. Here we further show that the enzyme activity of G6PDH in the infected shrimp hemocytes was promoted not only at 12 hpi but also at the earlier times of 0.5 and 6 hpi (Fig. 6). Again, this result suggests that the starting time of the WSSV-induced Warburg effect might be earlier than was previously thought, ie ~6 hpi instead of 12 hpi. Although most of the PPP intermediates were unaffected by WSSV infection (Fig. S4), downstream of the PPP, the levels of some of the precursors of purine and pyrimidine were increased (Fig. S5). These results suggest that in addition to elevating the levels of NADPH, the increased PPP metabolic throughput also elevated the levels of the nucleic acid precursors to biosynthesis. If all of these changes are being regulated by G6PDH, the inhibition of G6PDH should limit these effects: we found that chemical inhibition of this enzyme did in fact maintain the ROS at significantly higher levels at all time points (except 2 hpi, when the increase was not statistically significant; Fig. 7B) as well as significantly reduce virus production at 12 hpi in the cell (Fig. 7D).

In the glycolysis pathway, the levels of 3-PG and 2-PG were also elevated during WSSV infection (Fig. 5). Increased production of these two glycolytic intermediates causes glucose metabolism to be redirected into the serine and glycine biosynthesis pathway^27^. Diversion into this pathway is mostly driven by 3-PG, which is the branch point from glycolysis. The serine and glycine biosynthesis pathway is important for the production of the precursors for proteins, lipids, and nucleic acids, and activation of this pathway has been reported in many cancer cells^28^.

Our assays of the enzyme activities of PDH and LDH found that the activity of PDH, which converts pyruvate to acetyl-CoA, was reduced from 6 to 24 hpi while the activity of LDH, which converts pyruvate to lactic acid, remained unchanged (Fig. 6) Taken together, these changes suggest that pyruvate is not being efficiently metabolized by the TCA cycle. However, the activity of the TCA cycle was not completely abolished by the WSSV-induced Warburg effect (Fig. S7), which suggests that it might still be able to play an important role in anabolic metabolism during infection. Here, we observed that the levels of citrate were increased at 12 hpi even while other metabolites in the TCA cycle were unaffected (Fig. S7). In the mitochondria, citrate is produced from acetyl-CoA, and the citrate shuttle then exports this citrate into the cytosol, where it contributes to lipid and cholesterol synthesis^29^. However, as noted above, WSSV inhibits PK activity, and this in turn would reduce the amount of pyruvate available for conversion into acetyl-CoA via the TCA cycle. An alternative carbon source for acetyl-CoA synthesis is provided by glutamine metabolism. In glutaminolysis, glutamine is converted to glutamate, and at 12 hpi we found that while the levels of glutamate were increased, the levels of glutamine were unchanged (Fig. S7). Taken together, these results suggest that during WSSV infection, glutaminolysis but not glycolysis is being used to provide the alternative anapleurotic carbon source both for the TCA cycle’s energy production and for the lipid synthesis which is necessary for virus replication.

**Figure 8:**
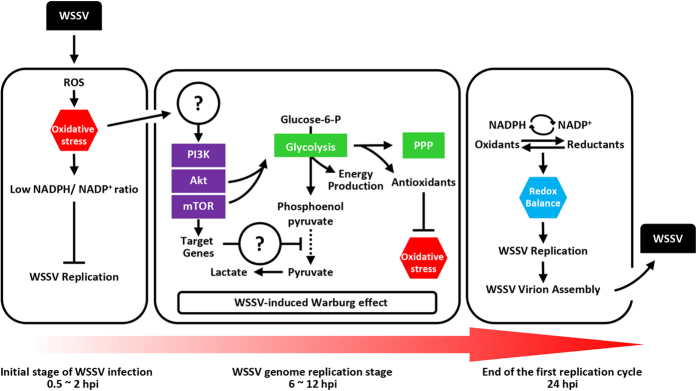
Schematic representation of how WSSV combats the host’s initial oxidative stress defenses by inducing metabolic changes in the genome replication stage through to the end of the first replication cycle.

The PI3K-Akt-mTOR pathway is known to be one of the critical regulators in cancer cells and proliferating cells. Dysregulation of this pathway causes a normal cell to becoming a cancer cell, and this leads not only to gene amplification and protein overexpression, but also to the characteristic signs of the Warburg effect^30^. In shrimp, two of the changes associated with the WSSV-induced Warburg effect, ie increased lactic acid levels and elevated glycolysis activity, were both suppressed when this pathway was entirely blocked^17^. The PI3K-Akt-mTOR pathway was also found to induce lipid biosynthesis to support WSSV morphogenesis^31^. Here, we used a PI3K-Akt-mTOR inhibitor to study another important aspect of the Warburg effect in WSSV pathogenesis. Based on the results shown in Fig. 7, we concluded that the PI3K-Akt-mTOR-regulated Warburg effect was acting to neutralize ROS production and reduce the host’s oxidative stress defenses during WSSV infection, thereby restoring the cell to a state of redox balance and allowing the virus to successfully replicate. This is illustrated graphically by the schematic presented in Fig. 8.

## Materials and Methods

### Experimental animals and virus inoculum

The adult, specific pathogen free (SPF) shrimp (*Penaeus vannamei*, body weight: 15 g) used in this study were obtained from the culture farm of Tungkang Biotechnology Research Center, Fisheries Research Institute, Pingtung, Taiwan. Before the experiments, the shrimp were acclimated in water tanks with a salinity of 35 ppm at 27 °C for 3 days. The virus used throughout the study was white spot syndrome virus (WSSV) (TW-1 isolate), which was originally isolated from naturally WSSV-infected *P. monodon* shrimp collected in 1994^32^. Virus stock was prepared as described previously^6^. Before use, it was thawed and further diluted with phosphate-buffered saline (PBS; 137 mM NaCl, 2.7 mM KCl, 10 mM Na_2_HPO_4_, 2 mM KH_2_PO_4_) to a final experimental inoculum titer that caused approximately 100% cumulative mortality in the adult SPF shrimp at 72 h post WSSV challenge. Shrimp injected with sterilized PBS served as control groups in this study. During the experiments, water salinity and temperature were as described above.

### Observation of intracellular reactive oxygen species (ROS) production by confocal spectral microscopy

Analysis of the ROS levels in shrimp hemocytes was conducted using an Image-iT Live Green Reaction Oxygen Species Detection kit (Invitrogen, USA) and a primary shrimp hemocyte culture system as described previously^6^. Briefly, shrimp hemolymph samples were collected from the control and WSSV-injected shrimp at various time points (0.5, 1, 2, 6, 12, 24 h post injection, hpi) using syringes containing an equal volume of shrimp anticoagulant solution (450 mM NaCl, 10 mM KCl, 10 mM EDTA, 10 mM Tris-HCl, pH 7.5). Subsequently, the hemocytes in the collected samples were incubated in culture medium (2x Leibovitz’s 15 medium [L-15; Invitrogen, USA], 10% fetal bovine serum [FBS], 1% glucose, 0.005% NaCl, 100 U/ml penicillin, 100 U/ml streptomycin, and 1.25 μg/ml amphotericin B [Fungizone]) with 25 μM carboxy-H_2_DCFDA on glass coverslips in the dark at room temperature for 1.5 h. This allows the H_2_DCFDA to passively diffuse into the hemocytes, where it is oxidized by cellular oxidative products and converted into DCF, which is a fluorescent product that is unable to permeate through the cell membrane. The medium was then replaced with fresh culture medium, and incubation proceeded for another 0.5 h. This was followed by specific counterstaining of the hemocyte nuclei with 1 μM Hoechst 33342 for 5 min. The hemocytes were then washed with PBS, fixed by 0.5% paraformaldehyde on ice for 10 min, and observed under a Leica TCS SP5 confocal spectral microscope. Higher quantities of ROS appeared as higher levels of green fluorescence.

### Determination of intracellular ROS production in shrimp hemocytes using flow cytometry

This method of ROS analysis also used the Image-iT Live Green Reaction Oxygen Species Detection kit (Invitrogen, USA). Shrimp hemolymph was collected as described above at 10 min and 30 min, and at 1, 2, 4, 6, 12, and 24 h post PBS and WSSV injection. Four pooled samples were prepared for each datapoint, with each pooled sample containing hemolymph from three shrimps. An equal volume of cooled PBS with 5 μM carboxy-H_2_DCFDA was added to each pooled sample. The samples were then placed on ice for 30 min, and protected from light to allow the H_2_DCFDA to permeate the hemocytes. A FACSCalibur flow cytometer (BD Biosciences, USA) was used to measure the fluorescence intensity of the carboxy-H_2_DCFDA treated shrimp hemocytes using techniques described in Brown and Wittwer^33^. The fluorescence intensity of each pooled sample was quantified from 30,000 cells, and results were expressed as the mean (n = 4) of the total ROS signal ± standard deviation (SD). Unpaired Student t tests were used to test for statistically significant differences between the WSSV-challenged shrimps and the corresponding PBS controls.

### Extraction of the metabolites from *P. vannamei*

Adult shrimps (*P. vannamei*) were randomly assigned to either the PBS control group or the WSSV challenge group. At 0, 0.5, 2, 6, 12, 18, and 24 hours post WSSV or PBS injection, 15 individuals were randomly selected from each group and five pooled hemolymph samples (each pooled sample containing hemolymph from three shrimp) were collected using a syringe containing an equal volume of cooled anticoagulant solution (PBS, 10 mM EDTA). The stomachs were also removed from the same shrimp, and similarly divided into five corresponding pooled samples. The pooled stomach samples were then stored immediately in liquid nitrogen. Each pooled hemolymph sample from above was then centrifuged at 800× g for 10 min. The resulting pellet was rinsed with cold PBS, carefully suspended with 100 μl of 0.33 x PBS and placed on ice for 10 min. In addition to being used for the extraction of hemolytic metabolites, these samples were also used for the relative quantification of the mRNA copy number of the WSSV genes. Both procedures are described in detail below.

Pleopods were also collected from each individual shrimp and used to quantify the WSSV genome copy number by real-time PCR as described below.

### Extraction of hemolytic metabolites

The hemocyte suspension was centrifuged at 14,000 × g for 10 min, and then 100 μl of lysate was transferred into a new tube, mixed with 200 μl of 100% methanol and centrifuged again at 14,000 × g for 10 min. The protein pellet was discarded. The supernatant was then transferred to a new tube and after being evaporated by a vacuum device, the metabolite-containing residue was stored at −20 °C.

### Extraction of lactic acid from shrimp plasma

Adult shrimps (*P. vannamei*) were randomly assigned to either the PBS control group or the WSSV challenge group. At 0, 0.5, 1, 2, 6, 12 and 24 hours post WSSV or PBS injection, 15 individuals were randomly selected from each group and five pooled hemolymph samples (each pooled sample containing hemolymph from three shrimp) were collected using a syringe containing an equal volume of cooled anticoagulant solution (PBS, 10 mM EDTA).

Each pooled hemolymph sample from above was then centrifuged at 800 × g for 10 min, and 200 μl of the plasma was mixed with 600 μl of 100% methanol and centrifuged at 14,000 × g for 10 minutes. The protein pellet was then discarded, while the supernatant was transferred to a new tube and evaporated by a vacuum device. The resulting lactic acid-containing residue was stored at −20 °C.

### Liquid-liquid extraction (LLE) of the polar metabolites from shrimp stomachs

Liquid-liquid extraction (LLE) is a method to separate compounds based on their relative solubilities in two different immiscible liquids, and we used this technique here to extract the redox molecules NADPH, NADP^+^, GSH, GSSG and the energy molecules NADH and NAD^+^ from shrimp stomachs. First, the pooled stomach samples preserved in liquid nitrogen were mixed with four times their weight of methanol. After the samples were cut into small pieces and homogenized in a bullet blender, the tissue lysate was centrifuged at 14,000 × g for 10 min. The supernatant (180 μl) was transferred to a fresh tube, mixed with an equal volume of chloroform by vigorous agitation, and centrifuged at 14,000 × g for 10 min to separate the two liquids. The upper methanol layer was transferred to a new tube for desiccation in a speed vacuum concentrator, and the resulting water-soluble polar metabolites were stored at −20 °C.

### Aniline derivatization of the hemocyte metabolites

To enhance the signal produced by the hemocyte metabolites, the residue extracted from the hemocytes was subjected to an aniline derivatization procedure, which results in the metabolites’ carbonyl, phosphophyl and carboxyl groups being labeled with aniline. The procedure was performed as described previously^34^ with some modifications. Briefly, each dry sample was dissolved in 35 μl of deionized water to which was added 5 μl of reaction buffer (0.3 M aniline [Sigma-Aldrich, USA] in 60 mM HCl) and 5 μl of freshly prepared N-(3-dimethylaminopropyl)-N′-ethylcarbodiimide hydrochloride (EDC; Sigma-Aldrich, USA) dissolved in deionized water to a concentration of 20 mg/ml. The mixture was vortexed and then incubated at ambient temperature (~26 °C) for 2 h. The reaction was stopped by adding 5 μl of 10% ammonium hydroxide.

### Liquid chromatography - electrospray ionization - mass spectrometry (LC-ESI-MS) of shrimp metabolites

The aniline-derivatized samples and the samples stored at −20 °C were analyzed using an LC-ESI-MS system consisting of an ultra-performance liquid chromatography (UPLC) system (Ultimate 3000 RSLC, Dionex) and a quadrupole time-of-flight (TOF) mass spectrometer with an electrospray ionization (ESI) source (maXis HUR-QToF system, Bruker Daltonics). The autosampler was set at 4 °C. Separation was performed by reversed-phase liquid chromatography (RPLC) on a BEH C18 column (2.1 × 100 mm, Walters). The elution started from 99% mobile phase A (0.1% formic acid in ddH_2_O) and 1% mobile phase B (0.1% formic acid in ACN). Mobile phase B was held at 1% for 0.5 min, raised to 60% over 6 min, further raised to 90% in another 0.5 min, held at 90% for 1.5 min, and then lowered back to 1% in 0.5 min. The column was then equilibrated by pumping 1% B for 4 min. The flow rate was set to 0.4 ml/min with an injection volume of 10 μl. The LC-ESI-MS chromatograms were acquired in negative ion mode under the following conditions: capillary voltage of 3,500 V, dry temperature at 190 °C, dry gas flow maintained at 8 l/min, nebulizer gas at 1.4 bar, and acquisition range of m/z 100–1,000.

The metabolite data were acquired by HyStar and micrOTOF control software (Bruker Daltonics) and processed by DataAnalysis and TargetAnalysis software (Bruker Daltonics). Each metabolite was identified as follows: first, the chemical composition of the metabolite was used to create a unique signature based on its theoretical mass over charge, its specific isotope pattern, and its predicted retention time. The LC-ESI-MS signals were then searched for a match against each signature. The amount of each metabolite was calculated from the area under the signal peaks in the ion chromatogram.

### Determination of glycolytic enzyme activities

PFK, PK, PDH, G6PDH and LDH activities were measured using a Phosphofructokinase Activity Colorimetric Assay Kit, a Pyruvate Kinase Activity Colorimetric/Fluorometric Assay Kit, a Pyruvate Dehydrogenase Activity Colorimetric Assay Kit, a Glucose-6-Phosphate Dehydrogenase Activity Colorimetric Assay Kit and a Lactate Dehydrogenase Activity Assay Kit respectively (BioVision, Inc., USA). Hemolymph samples were collected from WSSV- and PBS-injected shrimp at 0.5, 1, 2, 6, 12, and 24 hpi as described above. Hemocytes were collected from the hemolymph by centrifugation at 800 × g for 10 min. The cell pellets were suspended carefully with the assay buffer provided with the kits, and then placed on ice for 10 minutes. The suspensions were centrifuged again at 10,000 × g for 5 minutes, and the supernatant was retained and diluted into five equal portions for separate analysis by each of the five assay kits. Aliquots (50 μl) of the sample lysates were then placed into the wells of a 96 well plate with 50 μl of reaction mixture as provided by the respective kits. The absorbance of each sample at the appropriate wavelength was measured at T1 (0 min) by an absorbance reader (FlexStation 3 Benchtop Multi-Mode Microplate Reader, Molecular Devices, USA) to give the A1 value. The reactions were then incubated at room temperature for 10 min.

At T2 (10 min), the absorbance was measured again, to give the A2 value. The difference in absorbance, ΔA (A2-A1), was applied to the appropriate respective standard curve for each assay to obtain the B value (nmol) of the end product generated by each reaction. The enzyme activity was then calculated as follows: enzyme activity (mU/mg) = B/(ΔT × total protein), where ΔT = T2 − T1, and the total protein of each sample of lysate was measured using a Bio-Rad Protein Assay Kit (Bio-Rad, USA) according to the instructions provided by the manufacturer.

### Determination of ROS production in WSSV-infected shrimp that had been pretreated with a chemical inhibitor of the PI3K-Akt-mTOR signaling pathway

To study the role of the WSSV-induced Warburg effect in ROS production, the Warburg effect was suppressed by pretreating shrimp with the inhibitor LY294002 (0.625 μg LY294002/g shrimp) by intramuscular injection two hours before WSSV injection. The procedure was performed as described by Su *et al*.^17^. The LY294002 solution (BioVision, Inc., USA) was preserved in 10% DMSO and diluted with PBS before use. Shrimp that were pretreated with 0.01% PBS/DMSO vehicle only were used as controls. Shrimp hemolymph was collected as described above at 0.5, 2, 6, 12, and 24 h after PBS or WSSV injection. Four pooled samples were prepared for each datapoint, with each pooled sample containing hemolymph from three shrimps. The pooled hemolymph samples were subjected to flow cytometry to measure the ROS levels as described above. The pleopods from the same individuals were also collected for quantification of the WSSV genome copy numbers by real-time PCR.

### Determination of ROS production in WSSV-infected shrimp that had been pretreated with a chemical inhibitor of the pentose phosphate pathway

The pentose phosphate pathway was suppressed by pretreating shrimp with the inhibitor dehydroepiandrosterone, DHEA (1.25 μg DHEA/g shrimp)^35^, by intramuscular injection two hours before WSSV injection. The DHEA solution (BioVision, Inc., USA) was preserved in 10% DMSO and diluted with PBS before use. Shrimp hemolymph was collected as described above at 0.5, 2, 6, 12, and 24 h after PBS or WSSV injection. Shrimp that were pretreated with 0.01% PBS/DMSO vehicle only were used as controls. Shrimp that were collected for the 24 hpi time point were injected for a second time with DHEA or PBS/DMSO vehicle at 12 hours after the first injection. Four pooled samples were prepared for each datapoint, with each pooled sample containing hemolymph from three shrimps. The pooled hemolymph samples were subjected to flow cytometry to measure the ROS levels as described above. The pleopods from the same individuals were also collected for quantification of the WSSV genome copy numbers by real-time PCR.

### Absolute quantification of the number of copies of WSSV genomic DNA

The IQ REAL WSSV quantitative system (GeneReach, Taiwan) was used to quantify the absolute WSSV genomic DNA copy number in the pleopods of the LY294002-, DHEA- and PBS/DMSO- pretreated shrimp as well as the pleopods collected from the WSSV-infected shrimp in the metabolites study. Four pooled samples were prepared for each datapoint, with each pooled sample containing pleopods from three shrimps. After the shrimp DNA and the WSSV genomic DNA had been extracted using the DTAB/CTAB DNA extraction kit (GeneReach, Taiwan), the samples were analyzed on a real-time PCR system (ABI PRISM 7300, Applied Biosystems, USA) according to the instructions provided by the IQ REAL WSSV manual. A standard curve was generated from serial dilutions (10^6^, 10^5^, 10^4^, 10^3^, 10^2^, and 10^1^ copies/μl) of the Dual P (+) standard provided with the kit. The virion copy numbers were then calculated by reference to the Dual P (+) standard curve as described previously^6^.

### Relative quantification of the number of mRNA copies of three WSSV genes

The hemolymph samples prepared for the metabolite assays were also used to determine the number of *ie1, vp28*, and *icp11* mRNA copies in the infected shrimp hemocytes using real-time PCR. The shrimp hemocytes were collected as described above at 0, 6, 12, 18 and 24 h post WSSV injection. Five pooled samples were prepared for each datapoint, with each pooled sample containing hemocytes from three shrimps. The RNA was extracted from the pooled hemocytes, and the cDNA was synthesized by Superscriptase III reverse transcriptase (Invitrogen, USA) with the anchor dTv primer. Real-time PCR was performed with the primer sets EF1-α-qF/EF1-α-qR, ie1-qF/ie1-qR, vp28-qF/vp28-qR and icp11-qF/icp11-qR (Table 1) using a CFX384 Touch™ Real-Time PCR Detection System (Bio-Rad, USA) with KAPA SYBR^®^ FAST Universal 2X qPCR Master Mix (Kapa Biosystems, USA). The relative copy numbers were calculated by the 2^−Δ*CT*^ method as described by Wang *et al*.^36^.

## Additional Information

**How to cite this article**: Chen, I.-T. *et al*. Six Hours after Infection, the Metabolic Changes Induced by WSSV Neutralize the Host’s Oxidative Stress Defenses. *Sci. Rep.* **6**, 27732; doi: 10.1038/srep27732 (2016).

## Supplementary Material

Supplementary Information

## Figures and Tables

**Table 1:** Primer sequences used in this study.

Gene name	Primer sequences (5′–3′)	Usage
ie1	F : 5′-CAAGTACCCAGGCCCAGTGT-3′	qRT-PCR
	R : 5′- TGACCCACTCCATGGCCTT -3′	qRT-PCR
vp28	F : 5′- AGTTGGCACCTTTGTGTGTGGTA -3′	qRT-PCR
	F : 5′- TTTCCACCGGCGGTAGCT -3′	qRT-PCR
icp11	F : 5′- TTGAGGCAGTCAGGAAGAGTGA -3′	qRT-PCR
	F : 5′- GGCACACCATGTAAACACGGT -3′	qRT-PCR
EF-1α	F : 5′-TGCTCTGGACAACATCGAGC-3′	qRT-PCR
	F : 5′-CGGGCACTGTTCCAATACCT-3′	qRT-PCR
