# Supplementary material for: Six Hours after Infection, the Metabolic Changes Induced by WSSV Neutralize the Host’s Oxidative Stress Defenses
Source: Sci Rep. 2016 Jun 9;6:27732. doi: 10.1038/srep27732 (PMC4899751; doi:10.1038/srep27732)
Supplement: Supplementary Information [file srep27732-s1.pdf]

**Supplemental Data**

# Six Hours after Infection, the Metabolic Changes Induced by WSSV Neutralize the Host's Oxidative Stress Defenses

I-Tung Chen[a,b,1], Der-Yen Lee[c,d,1], Yun-Tzu Huang[a], Guang-Hsiung Kou[b], Han-Ching Wang[e*], Geen-Dong Chang[f,g*], and Chu-Fang Lo[a,b*]

[a]Institute of Bioinformatics and Biosignal Transduction, College of Bioscience and Biotechnology, National Cheng Kung University, Tainan 701, Taiwan

[b]Department of Life Science, National Taiwan University, Taipei 104, Taiwan

[c]Technology Commons, National Taiwan University, Taipei 106, Taiwan

[d]Department of Applied Chemistry, National Chiayi University, Chiayi 600, Taiwan

[e]Institute of Biotechnology, College of Bioscience and Biotechnology, National Cheng Kung University, Tainan 701, Taiwan

[f]Graduate Institute of Biochemical Sciences, National Taiwan University, Taipei 106, Taiwan

[g]Center for Systems Biology, National Taiwan University, No. 1, Section 4, Roosevelt Road, Taipei 106, Taiwan

## Optimizing and checking the *in vivo* shrimp metabolomics platform

In our LC-ESI-MS platform, an aniline derivatization reaction was used to label the metabolites. The aniline labeling was mediated by N-(3-dimethylaminopropyl)-N′-ethylcarbodiimide hydrochloride (EDC), which binds directly to the carbonyl, phosphophyl, and carboxyl groups on the metabolites (eg fumaric acid, malic acid, pyruvate acid) [34]. This technique significantly improved the LC separation and ESI efficiency (see Fig S1), and also attenuated the effect of ion suppression. The total number detected metabolites was generally increased by 50% in the aniline derivatized samples compared to the crude metabolite samples (data not shown).

This labeled metabolite LC-ESI-MS platform identified approximately 100 metabolites in shrimp hemocyte samples (n = 4~6) collected at 0, 6, 12, 18, 24 hpi from WSSV-infected and uninfected (PBS) shrimp (*P. vannamei*). A principle component analysis (PCA) [37] showed that the metabolomic profiles of WSSV-infected and uninfected samples at each time point formed distinct clades (Fig S2).

## WSSV promotes the synthesis of purine and pyrimidine precursors at the viral replication stage

Rerouting of glycolysis into the pentose phosphate pathway (PPP) is another characteristic of the Warburg effect. Here we found that the levels of four PPP

intermediates - ribose 5-phosphate, erythrose 4-phosphate, sedoheptulose 7-phosphate, and ribose - were mostly unaffected after WSSV infection (Fig S4). However, downstream of the PPP, we found increased intracellular levels of some of the precursors of purine and pyrimidine (Fig S5). This suggests that even though there was no accumulation of the PPP intermediates, there was probably nevertheless an increase in the throughput of this pathway.

**Replication of WSSV DNA occurs immediately after the synthesis of the purine and pyrimidine precursors**

The replication status of WSSV in the infected shrimp was monitored using the IQ REAL WSSV quantitative system. Absolute quantification of the number of copies of WSSV viral DNA in the pleopods of the infected shrimp showed that the accumulated WSSV DNA was initially very low. However, between 12 and 18 hpi, virus quantities rapidly increased from ~ 2.7 WSSV copies to ~424.8 WSSV genome copies per $10^4$ host genome copies (~157.3 times) (Fig S6A). Subsequently, between 18 and 24 hpi, the virus replication activity increased at a much slower rate (~1.9 times) (Fig S6A). Meanwhile, the increase in the number of WSSV *ie1*, *vp28*, and *icp11* genes in the WSSV-infected shrimp hemocytes also reached a peak between 12 and 18 hpi (Fig S6B~D). These results show that during the first WSSV replication cycle (~24 hpi), the

viral DNA replication stage immediately followed the synthesis of the purine and pyrimidine precursors (Fig S5).

**TCA cycle metabolism was not abolished during WSSV infection despite the accumulation of lactic acid**

During the aerobic glycolysis observed in the Warburg effect, glucose is mostly converted to lactic acid instead of being catabolized by the TCA cycle. Here, however, despite the production of large amounts of lactic acid during WSSV infection (Fig 4A), the relatively small changes in the levels of the TCA metabolites (Fig S7) suggested that the TCA cycle was still functioning. We note, however, that the higher levels of citrate and the reduced levels of succinate indicate that the TCA cycle might have been diverted into citrate cataplerosis as well as replenished by glutamine/ glutamate anaplerosis.

# SUPPLEMENTAL FIGURES

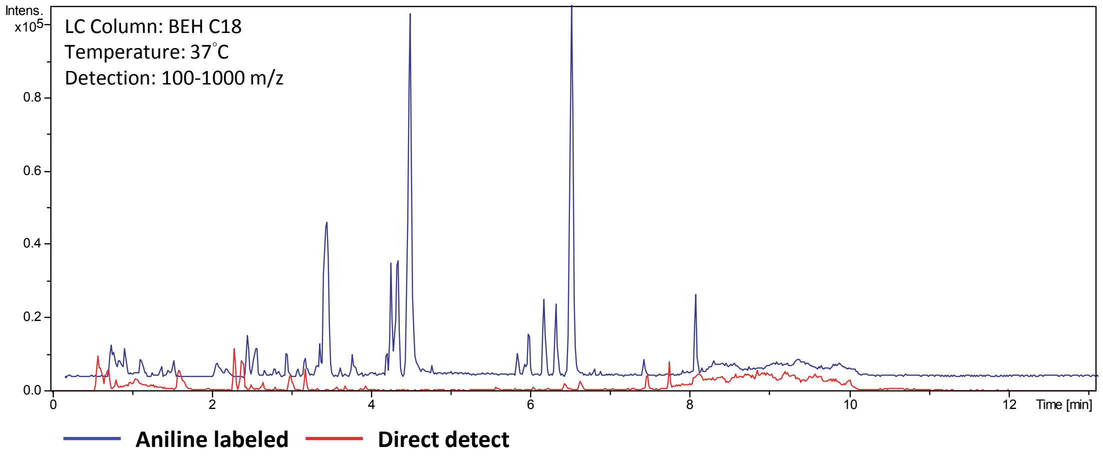


**Fig S1.**

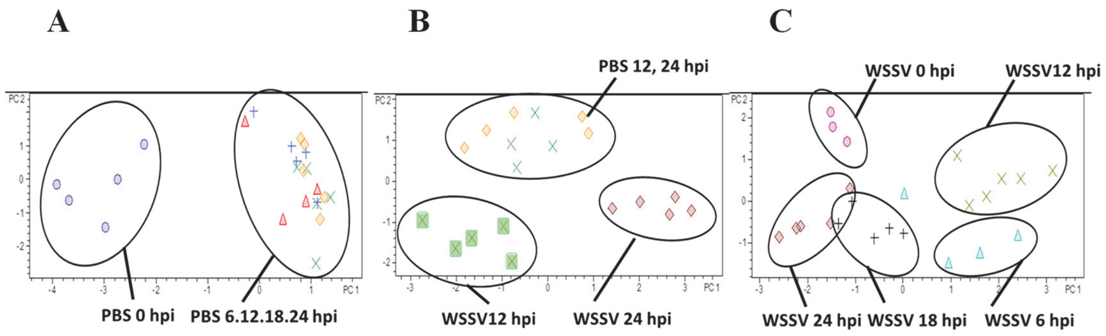
A
B
C
PBS 0 hpi
PBS 6.12.18.24 hpi
PBS 12, 24 hpi
WSSV12 hpi
WSSV 24 hpi
WSSV 0 hpi
WSSV12 hpi
WSSV 24 hpi
WSSV 18 hpi
WSSV 6 hpi

**Fig S2.**

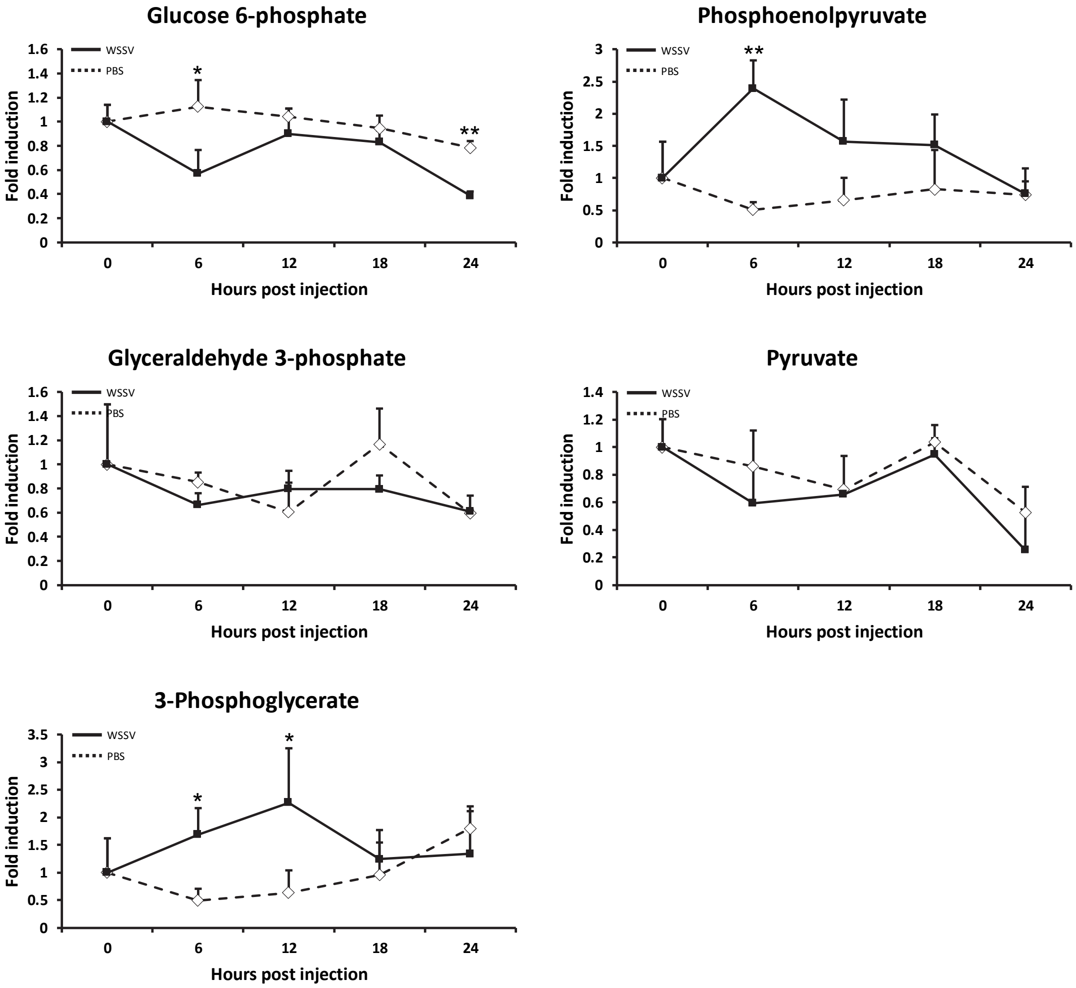

Glucose 6-phosphate
Phosphoenolpyruvate
Glyceraldehyde 3-phosphate
Pyruvate
3-Phosphoglycerate
WSSV
PBS
Fold induction
Hours post injection
0
6
12
18
24
*
**


**Fig S3.**

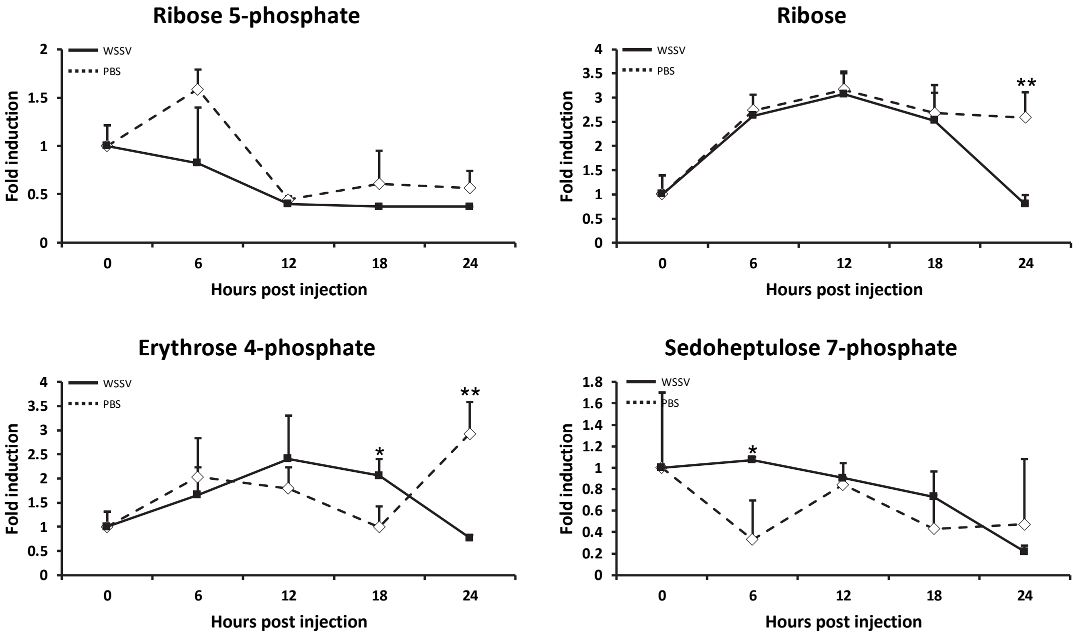
Ribose 5-phosphate
Ribose
Erythrose 4-phosphate
Sedoheptulose 7-phosphate
WSSV
PBS
Fold induction
Hours post injection
0
6
12
18
24
*
**

**Fig S4.**

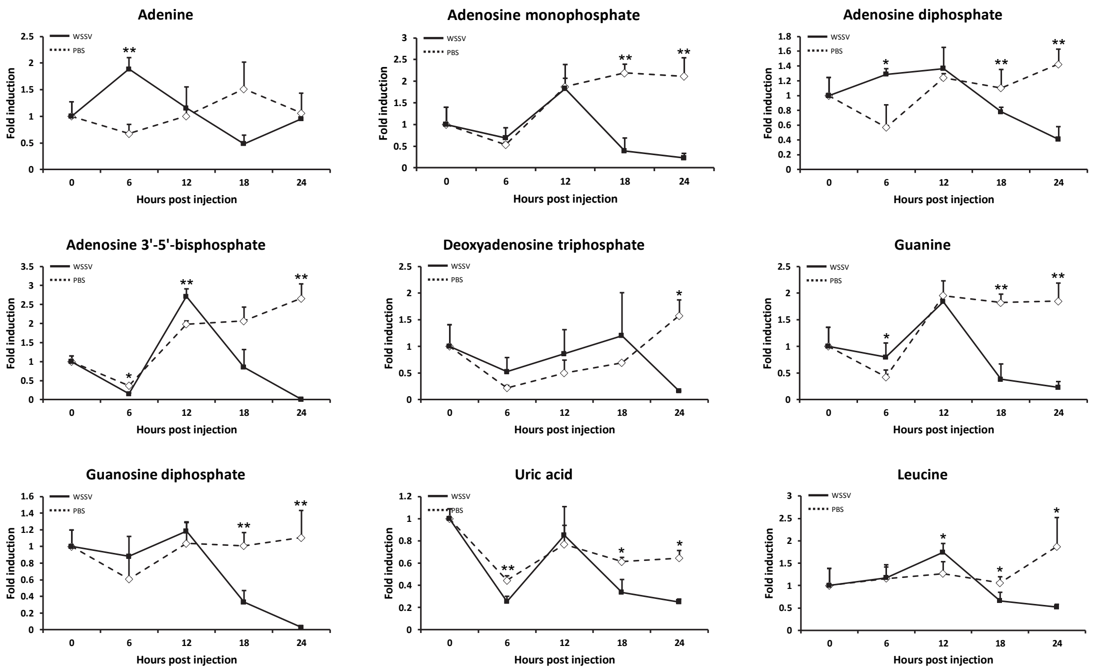
Adenine
Adenosine monophosphate
Adenosine diphosphate
Adenosine 3'-5'-bisphosphate
Deoxyadenosine triphosphate
Guanine
Guanosine diphosphate
Uric acid
Leucine
WSSV
PBS
Fold induction
Hours post injection

**Fig S5.**

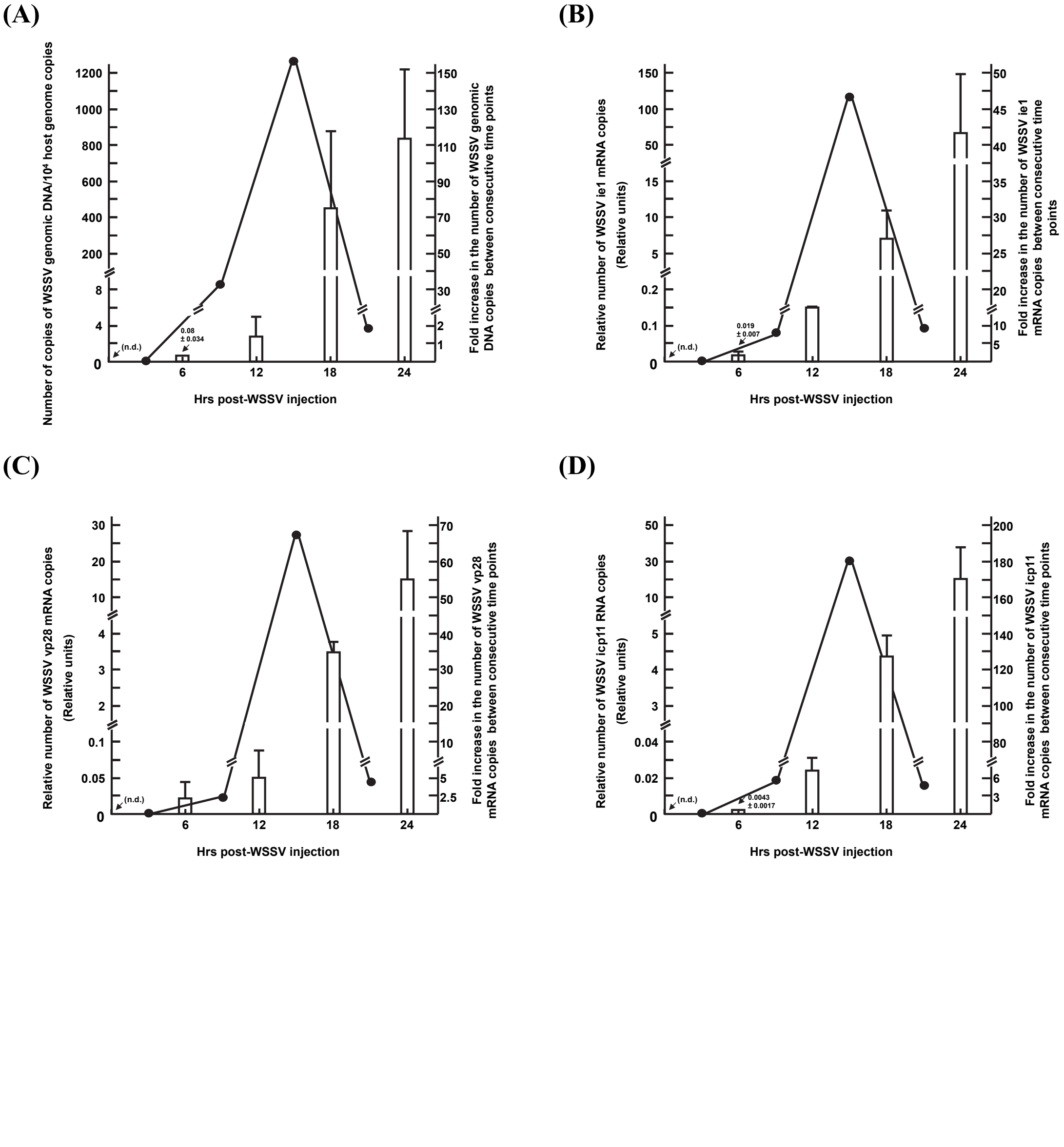
(A)
Number of copies of WSSV genomic DNA/10⁴ host genome copies
Fold increase in the number of WSSV genomic DNA copies between consecutive time points
(n.d.)
0.08 ± 0.034
Hrs post-WSSV injection
(B)
Relative number of WSSV ie1 mRNA copies (Relative units)
Fold increase in the number of WSSV ie1 mRNA copies between consecutive time points
(n.d.)
0.019 ± 0.007
Hrs post-WSSV injection
(C)
Relative number of WSSV vp28 mRNA copies (Relative units)
Fold increase in the number of WSSV vp28 mRNA copies between consecutive time points
(n.d.)
Hrs post-WSSV injection
(D)
Relative number of WSSV icp11 RNA copies (Relative units)
Fold increase in the number of WSSV icp11 mRNA copies between consecutive time points
(n.d.)
0.0043 ± 0.0017
Hrs post-WSSV injection

**Fig S6.**

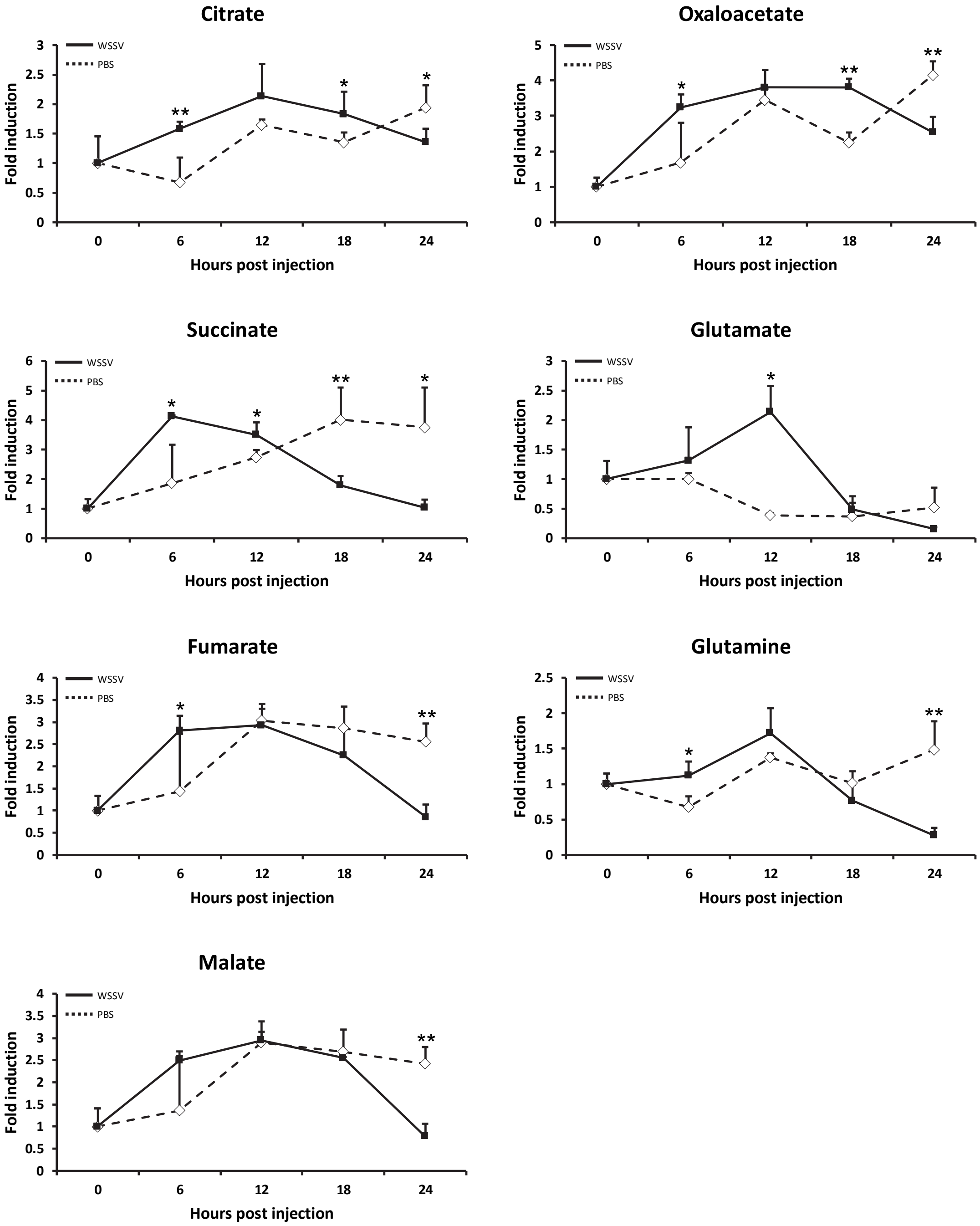
Citrate
Oxaloacetate
Succinate
Glutamate
Fumarate
Glutamine
Malate
WSSV
PBS
Fold induction
Hours post injection
0
6
12
18
24
*
**

**Fig S7.**

# SUPPLEMENTAL FIGURE LEGENDS

Fig S1. LC-ESI-MS spectra showing improved LC separation and efficiency after aniline labeling.

Fig S2. Principle Component Analysis (PCA) showing the distinct clades formed by the metabolites of hemocytes collected from (A) PBS-injected shrimp, (B) WSSV- vs. PBS-injected shrimp at 12 and 24 hpi, and (C) WSSV-injected shrimp at 0-24hpi

Fig S3. Measurement of the levels of five glycolytic metabolites in the stomachs of WSSV-infected shrimp. Each bar shows the mean ± SD level of the indicated glycolytic metabolite relative to its level at 0 hpi. Single ($P<0.05$) and double ($P<0.005$) asterisks indicate a statistically significant difference between the experimental and control groups.

Fig S4. Measurement of the levels of four PPP metabolites in the hemocytes of WSSV-infected shrimp. Each bar shows the mean ± SD of the indicated pentose phosphate pathway metabolite relative to its level at 0 hpi. Single ($P<0.05$) and double ($P<0.005$) asterisks indicate a statistically significant difference between the experimental and

control groups.

Fig S5. The effect of WSSV infection on the profiles of metabolic intermediates involved in the synthesis of purine and pyrimidine precursors. Each bar shows changes in the mean ± SD of metabolites involved in the synthesis of purine and pyrimidine precursors relative to their respective levels at 0 hpi. Single (P<0.05) and double (P<0.005) asterisks indicate a statistically significant difference between the experimental and control groups.

Fig S6. WSSV genome replication and gene expression status in shrimp. (A) Each bar represents the number of copies of WSSV genomic DNA in pooled pleopod samples. (B-D) The bars represent the relative levels of (B) WSSV *ie1* mRNA, (C) WSSV *vp28* mRNA and (D) WSSV *icp11* mRNA detected in pooled hemocytes samples at the indicated time points. The curves show the relative change in copy number or gene expression from one time point ($T_n$) to the next ($T_{n+1}$) as defined by ($T_{n+1}$)/ ($T_n$).

Fig S7. The effect of WSSV infection on the profiles of metabolic intermediates in the TCA cycle. Each bar shows the mean ± SD of the indicated tricarboxylic acid cycle (TCA) metabolite relative to its level at 0 hpi. Single (P<0.05) and double (P<0.005)

asterisks indicate a statistically significant difference between the experimental and control groups.

## SUPPLEMENTARY REFERENCE

37. Yeung, K. Y. & Ruzzo, W.L. Principal component analysis for clustering gene expression data. *Bioinformatics*. **17**: 763-774 (2001).
